# Dexamethasone and postoperative analgesia in minimally invasive thoracic surgery: a retrospective cohort study

**DOI:** 10.1186/s44158-021-00023-6

**Published:** 2021-12-10

**Authors:** Marzia Umari, Giacomo Paluzzano, Matteo Stella, Valentina Carpanese, Giovanna Gallas, Caterina Peratoner, Giulia Colussi, Gaia Maria Baldo, Edoardo Moro, Umberto Lucangelo, Giorgio Berlot

**Affiliations:** Department of Anesthesia and Intensive Care, Azienda Sanitaria Universitaria Giuliano-Isontina, Cattinara University Hospital, Trieste, Italy

**Keywords:** Dexamethasone, Postoperative pain, Minimally invasive thoracic surgery, Multimodal analgesia

## Abstract

**Background:**

Dexamethasone is commonly used for the prevention of postoperative nausea and vomiting (PONV), and recent reviews suggest a role for dexamethasone in postoperative analgesia. The aim of this study is to evaluate the efficacy of dexamethasone as an analgesic adjuvant in minimally invasive thoracic surgery. Primary outcome was morphine consumption 24 h after surgery; secondary outcomes were pain control, measured as numeric rating scale (NRS), glycemic changes, PONV, and surgical wound infection.

**Results:**

We performed a retrospective cohort study considering 70 patients who underwent elective lobectomy, segmentectomy, or wedge resection surgery with a mini-thoracotomy approach or video-assisted thoracoscopic surgery (VATS). All patients received the same locoregional techniques and short-acting opioids during surgery; 46 patients received dexamethasone at induction. There were no significant differences in morphine consumption at 24 h (*p* = 0.09) and in postoperative pain scores. Nevertheless, a higher frequency of rescue therapy (*p* = 0.01) and a tendency for a higher attempted-PCA pushes count were observed in patients who did not receive dexamethasone. No cases of surgical wound infections were detected, and the incidence of PONV was similar in the two groups. Postoperative glycemia was transiently higher in the dexamethasone group (*p* = 0.004), but the need of hypoglycemic therapy was not significantly different.

**Conclusions:**

Preoperative administration of dexamethasone did not cause a significant reduction in morphine consumption, but appears to be safe and plays a role in a multimodal anesthesia approach for patients undergoing elective minimally invasive thoracic surgery.

## Background

An appropriate acute pain management after thoracic surgery is crucial to reduce postoperative respiratory complications, to facilitate recovery, rehabilitation, and patient satisfaction [1, 2]. Multimodal analgesic strategies aim to minimize the adverse effects of each drug administered, while achieving optimal pain control [3]. Dexamethasone is a drug commonly used in anesthesia for the prevention of *postoperative nausea and vomiting* (PONV) [4–7] and is used as an adjuvant to peripheral nerve block, since it has shown to prolong duration of sensory block with either perineural or intravenous administration [8, 9].

Many studies demonstrated that patients receiving dexamethasone in the perioperative period experienced less pain, requested a minor dosage of opioids in the postoperative period, needed fewer rescue doses, and had a shorter stay in post-anesthesia care units as well [10]. A recent review shows that 8 mg of dexamethasone before surgical incision may be beneficial in some surgeries, such as laparoscopic cholecystectomies, thyroidectomies, laparoscopic gynecologic surgery, and breast surgery, but there is no unequivocal result for each specialty [11]; doses greater than 8 mg did not show further analgesic effectiveness or reduction in opioid use [12, 13].

Regarding perioperative dexamethasone administration, the immunosuppressive effect and the hyperglycemia are major concerns since both can increase the risk of infections. Studies have demonstrated that there is no association between perioperative dexamethasone and incidence of surgical site infections (SSI) or delay in wound healing [10, 14]. Randomized trials failed to prove a significant alteration of glucose homeostasis either in diabetic or in non-diabetic patients, and although a hyperglycemic state after dexamethasone appeared to extend to the first 24 h after surgical operation, blood levels remained in the recommended range during the perioperative period [15].

The goal of the present study was to evaluate the analgesic properties of the administration of intravenous dexamethasone and the associated postoperative complications, such as wound infections and hyperglycemia, in patients who underwent lung resection surgery with a mini-thoracotomy approach or *video-assisted thoracoscopic surgery* (VATS).

## Methods

### Design

A retrospective cohort study was performed in patients who underwent minimally invasive thoracic surgery from January 10, 2017, to November 6, 2019, at the University Hospital of Trieste, Italy (NCT04325984). Ethical committee approved the study (CEUR-2019-Os-03) and written informed consent was obtained from all subjects. Perioperative patients’ data were collected from an ongoing Registry in use in our Institution (POPARTS Study- Post-Operative Pain After Recovery in Thoracic Surgery: Evaluation of the Persistence of Painful Symptoms and the Incidence of Neuropathic Pain After Resective Lung Surgery).

Patients were divided in two groups: in “Dexamethasone group” were included patients who received dexamethasone 8 mg prior to anesthesia induction and in “Control group” patients who received ondansetron 4 mg before extubation.

### Inclusion criteria

Patients were eligible if they were 18 years of age or older, ASA I-III, and had a BMI < 30 kg/m^2^. Written informed consent was obtained, and in order to reduce population heterogeneity, patients were included if they had been treated with the same anesthetic protocol.

### Perioperative management

Ultrasound-guided serratus anterior plane block (SAPb) was performed 30 min before surgical incision; in addition to it, paravertebral block (PVB) was performed by the surgeon under direct vision, at the end of surgery. Balanced anesthesia was performed with inhalatory sevoflurane and intravenous remifentanil. PONV prophylaxis was achieved either with dexamethasone 8 mg administered before induction or with ondansetron 4 mg administered 30 min before extubation. In patients with higher PONV risk with an Apfel score > 2, droperidol 0.625 mg was added, according to institutional protocol. During the perioperative period, glycemic values were kept between 70 and 180 mg/dl according to local protocol. Intravenous morphine bolus and 1 g paracetamol bolus were administered at least 30 min before the end of the surgery. Intraoperative morphine boluses varied according to surgery type. All patients, once fully awake, received an intravenous patient-controlled analgesia (PCA) device delivering morphine that allowed the self-administration of a maximum of 4 boluses of 1 mg per hour, with a lock-out interval of 10 min between boluses; maximum boluses were reduced to 2 per hours during the night. In case of poorly controlled pain, a basal morphine infusion was added. All patients received scheduled paracetamol and non-steroidal anti-inflammatory drugs (NSAIDs) were given as rescue therapy, when needed. Blood analysis was sampled after surgery and in the next morning, according to institutional protocol.

### Exclusion criteria

Exclusion criteria were inability to give informed consent, chronic therapy with medium-high doses of corticosteroids, chronic therapy with opioids, METS ≤4, urgent or emergency surgery, kidney failure at stage III or higher, liver failure, pregnancy, drug addiction, or history of drug abuse.

All patients were extubated in the operative room and then monitored for a couple of hours in the recovery room before going back to the ward.

### Endpoints

The primary endpoint was cumulative morphine consumption 24 h after surgery; morphine consumption was calculated as total morphine equivalents, normalized by adjusted body weight (ABW) calculated as ideal body weight (IBW) + 0.4*(total body weight − IBW). Secondary outcomes were static and dynamic NRS for pain, glycemia, and need of intraoperative hypoglycemic therapy during the first 24 h after surgery, incidence of PONV, and incidence of surgical wound infection.

### Data collection

Pain score was assigned using a numeric rating scale, by which patients were asked to rate their pain on a scale from 0 (no pain) to 10 (disabling pain) at resting position, while moving and while coughing. Pain score assessments were made 1 and 24 h postoperatively. At the same time points analgesic rescue doses and PONV were reported. Number of pushes and total morphine administration were calculated from PCA records. Surgical wound infections, reported at hospital discharge or during the first surgical follow-up, were recorded.

### Statistical analysis

The estimated minimum number of participants was 70 patients and sample size calculation was based on result on a previous study conducted in laparoscopic surgery [16] considering *α* = 0.05 and *β* = 0.2.

Independence between treatment and nonparametric variables was assessed by the chi-squared test. Normality was tested with Shapiro-Wilk test. Because of non-normal distribution observed, the Wilcoxon rank sum test was used to compare the distributions in the pain scores, morphine administration, and blood glucose values between the dexamethasone and the control group. Linear regressions were used to test associations between perioperative factors and morphine consumption. Multiple linear regression with Poisson distribution was used to test associations between perioperative factors and PCA pushes count at 24 h. Statistical analyses were conducted using R version 3.6.3.

## Results

A total of 70 patients were included in the analyses and 46 (66%) of them received preoperative dexamethasone; the others 24 (34%) were used as a control group. There were not relevant differences in patient baseline characteristic and surgical procedure between the group of patients who received dexamethasone and the control group (Table 1).

**Table 1:** Patient demographics and intraoperative characteristics

	Dexamethasone	Control
	(*n* = 46)	(*n* = 24)
Sex		
Female	14	12
Male	32	12
Age (years)*	66.3 (10.8)	66.9 (11.2)
Weight (kg)*	75.0 (12.0)	69.2 (16.6)
BMI*	25.3 (3.8)	24.3 (3.9)
Surgery type		
VATS	24	11
Minimally invasive thoracotomy	22	13
Intervention		
Wedge resection and segmentectomy	23	10
Lobectomy	23	14
APFEL score		
1	15	3
2	22	12
3	9	9
Diabetes mellitus	7	6
Morphine bolus (mg/kg ABW)*	0.15 (0.03)	0.16 (0.02)

Values are absolute count or *means (standard deviation)

Intraoperative morphine bolus was similar for the two groups, also when different surgical approaches and interventions were taken into consideration as confounding factors in a multivariate analysis (0.15 mg/kg ABW in the dexamethasone group vs 0.16 mg/kg ABW in the control group, *p* = 0.31).

Pain was well controlled in both groups, without significant differences between groups (Table 2).

**Table 2:** Pain scores variables

	Dexamethasone	Control	*p* value
	(*n* = 46)	(*n* = 24)	
Static NRS			
1 h	1.89	1.83	0.65
24 h	1.59	1.67	0.13
Dynamic NRS			
1 h	2.49	2.67	0.82
24 h	2.69	2.58	0.76
Cough NRS			
1 h	3.11	3.21	0.98
24 h	3.63	3.54	0.56

Values are means; *p* values are calculated with Wilcoxon rank-sum test

Total postoperative morphine consumption attempted and administered PCA pushes at 1 and 24 h after surgery did not differ between groups (Table 3).

**Table 3:** Morphine consumption and rescue therapies

	Dexamethasone	Control	*p* value
	(*n* = 46)	(*n* = 24)	
Morphine equivalents (mg/kg ABW)			
1 h	0.02	0.03	0.19
24 h	0.23	0.32	0.17
Attempted PCA pushes—24 h	24.8	27.6	0.32
Administered PCA pushes—24 h	14.7	14.5	0.94
NSAIDs*	9	6	0.6
Continuous morphine infusion*	0	3	0.01

Values are means or *absolute count; *p* values are calculated with Wilcoxon rank-sum test or *chi-squared test

**Fig. 1:**
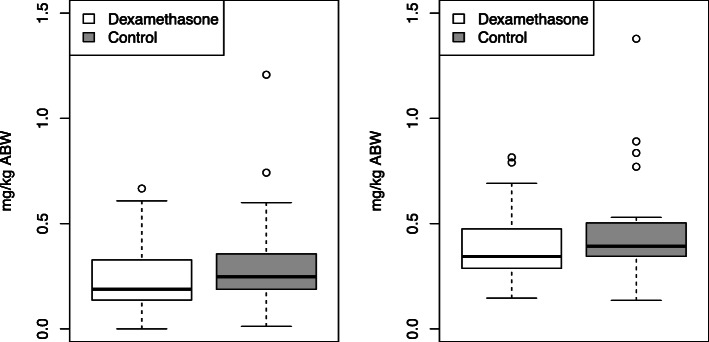
Morphine consumption at 24 h in the dexamethasone and the control group. The first plot shows postoperative morphine consumption. The second plot shows the sum of intraoperative and postoperative morphine consumption

Moreover, there were no differences in the two groups regarding the total amount of morphine administered postoperative and intraoperative (Fig. 1).

No strong association was found between dexamethasone administration and postoperative morphine consumption with univariate model (*r* = −0.083, *p* = 0.093). To exclude the influence of surgical approach and type of intervention on calculations, multiple linear regression was computed (Table 4).

**Table 4:** Multivariate analysis on morphine consumption and PCA pushes

	Dexamethasone	Intervention	Surgery type
	*r* (*p*-value)	*r* (*p*-value)	*r* (*p*-value)
Morphine equivalents—24 h	− 0.086 (0.086)	0.0039 (0.936)	0.0425 (0.375)
Erogated PCA pushes—24 h	0.011 (0.866)	0.102 (0.111)	− 0.080 (0.212)
Attempted PCA pushes—24 h	− 0.095 (0.054)	0.096 (0.046)	− 0.304 (< 0.001)

Regression coefficients (*p* values)

The analysis failed to find a statistically significant association between the administration of dexamethasone in the studied cohort and morphine consumption at 24 h after surgery (*p* = 0.086). Likewise, the same calculation was done on attempted and administered PCA pushes in the first 24 h, based on the idea that PCA bolus doses can be considered a surrogate of a patient’s pain sensation over time. Attempted pushes count was generally higher since it included erogated pushes plus pushes without morphine delivery by PCA device due to lockout or max hourly dose. Also in this case no significant association was noticed in PCA erogated pushes over the first 24 postoperative hours (*p* = 0.866). Interestingly, considering all attempted PCA pushes, a tendency of lower morphine demand was observed in patients who received dexamethasone (*p* = 0.054), as well as statistically significant differences based on the different intervention (wedge resection vs lobectomy) or surgical type (VATS vs minithoracotomy) (Table 4).

NSAIDs administration was comparable between the two groups; however, in 3 patients of the control group, a rescue basal morphine infusion was settled by the Acute Pain Service team due to poor pain control. On the other side, no patient of the dexamethasone group needed a change in PCA settings (*p* = 0.01).

There was a significant increase in postoperative glycemia in patients who received dexamethasone compared to the control group (*p* = 0.004); anyway the difference was not statistically relevant at 24 h after surgery (Table 5).

**Table 5:** Perioperative glycemic variations

Glycemia (mg/dL)	Dexamethasone		Control		*p* value
	(*n* = 46)		(*n* = 24)		
Baseline	103 [96–112.8]		98.5 [92.5–116]		0.47
1 h	154 [137–168]	(+ 49.5%)	128 [120–154.5]	(+ 29.9%)	0.004
24 h	124 [106.5–134.8]	(+ 20.9%)	106 [96.5–132.5]	(+ 7.6%)	0.07

Values are medians [interquartile range] (% variation from baseline); *p* values are calculated with Wilcoxon rank-sum test

Moreover, clinical relevance of observed difference was scarce and the need for perioperative hypoglycemic therapy (when glycemia > 180 mg/dL) was low and did not differ significantly between the two groups (*p* = 0.22). PONV incidence was similar in the two groups (*p* = 0.49) and no cases of surgical wound infection were observed.

## Discussion

Our data show that perioperative administration of a single dose of dexamethasone seems not associated with a significant reduction in morphine consumption after minimally invasive thoracic surgery. Nevertheless, dexamethasone administration appeared to be safe and easily integrated in a multimodal analgesia approach [17]. No side effects were observed in the intraoperative and postoperative period, especially no case of wound infection or impaired healing was reported from the surgeon, and this is consistent with ongoing literature [14]. All patients received a multimodal anesthetic management with a standardized locoregional approach consisting of both SAPb and PVB. In the postoperative period, patients were first moved to a recovery room and then transferred to the ward, where Acute Pain Service was actively involved. All these factors contributed to achieve satisfactory pain management in both groups, as this is reflected from the low average NRS among patients (Table 2).

Despite the comparable overall morphine consumption between the two groups, a higher frequency of rescue medications was observed in patients who did not receive dexamethasone and that required a continuous morphine infusion. In fact, while patients who received dexamethasone did not require any significant change in the postoperative analgesic plan, three patients of the control group required an additional basal morphine infusion to achieve adequate pain control (Table 3). This anyway did not end up in a significant total morphine consumption between the two groups that was the primary endpoint of this study. PCA is commonly used to optimize morphine postoperative morphine consumption and avoid overtreatment, thus minimizing opioid side effects and pulmonary complications. It also allows early postoperative mobilization and physiotherapy [18]. Furthermore, PCA attempted pushes can also work as an index of pain sensation over time. Applying a multivariate analysis on PCA attempted pushes count a small but significant difference was observed among patients that received a different intervention (wedge resection vs lobectomy) or a different surgical approach (VATS vs mini-thoracotomy). A tendency in a higher PCA attempted pushes count was observed also in the group of patients who did not receive dexamethasone, yet this finding lacks statistical significance (Table 4). On the other side, this study confirmed that a single preoperative dexamethasone administration leads to no additive adverse effects. No wound infection and/or delayed healing associated with dexamethasone use was observed. Furthermore, PONV incidence in the dexamethasone group was similar, when compared to the control group, where 5-HT antagonists were used. A transient hyperglycemia was observed after dexamethasone administration; nevertheless, blood levels remained in the recommended perioperative range, leading to a similar need for hypoglycemic therapy in the two groups, and no more statistically differences in glycemia were found after 24 h (Table 5). In this study, a single dose of dexamethasone was tested and the possible effects of higher doses of dexamethasone were not investigated. Anyway, a standard dose of 8 mg dexamethasone is approved by ERAS protocols [19] and nowadays generally accepted as analgesic adjuvant dose, while doses greater than 8 mg did not show further analgesic effectiveness or reduction in opioid use [12, 13]. One possible explanation of the lack of significatively in morphine consumption might be found in the peculiar pathophysiology of post thoracotomy pain. Pain after thoracotomy arises from both nociceptive and neuropathic mechanisms which may originate from somatic and visceral afferents [20]. This could make the analgesic effect of dexamethasone less influential, when compared to other kinds of surgery, characterized by a pain of lesser extent, or with pathophysiologic features, like for example odontoiatric and orthopedic surgery, where the anti-inflammatory role of dexamethasone could be emphasized. In addition, in this study both VATS and mini-thoracotomy approaches were included, while in recent literature VATS approach has been linked to improved postoperative respiratory function, reduced hospital length of stay, and a higher level of tolerability for the patients compared to thoracotomy [21].

The main limitation of the present study was its retrospective nature. The two groups were not well balanced with more patients receiving dexamethasone than controls and this reflected the increasing trend to include dexamethasone in the clinical practice in the last years. Anyway, sample size calculation was calculated from the hypothesis of balanced population. Surgical approaches and invasiveness of resection were heterogeneous in the population studied. In the study, a single dose of dexamethasone was tested, avoiding looking for a dose–response relationship. Finally, PCA settings were independent from sex and patients’ body weight and could have been too restrictive, hiding the effect of dexamethasone in the primary outcome.

## Conclusions

No significant difference was observed in overall morphine consumption 24 h after surgery. Despite this, a lower recall in rescue analgesia was noticed in the dexamethasone group and no evident side effects were related to dexamethasone usage. Further studies are required to evaluate the effectiveness of the drug and to confirm the absence of increase of long-term postoperative complications.

## Data Availability

The datasets used and analyzed during the current study are available from the corresponding author on reasonable request.

## References

[1] Wenk M, Schug SA (2011). Perioperative pain management after thoracotomy. Curr Opin Anaesthesiol.

[2] Doan LV, Augustus J, Androphy R, Schechter D, Gharibo C (2014). Mitigating the impact of acute and chronic post-thoracotomy pain. J Cardiothorac Vasc Anesth.

[3] Chen JQ, Wu Z, Wen LY, Miao JZ, Hu YM, Xue R (2015). Preoperative and postoperative analgesic techniques in the treatment of patients undergoing transabdominal hysterectomy: a preliminary randomized trial. BMC Anesthesiol.

[4] Henzi I, Walder B, Tramèr MR (2000). Dexamethasone for the prevention of postoperative nausea and vomiting: a quantitative systematic review. Anesth Analg.

[5] Sinner B (2019). Perioperatives Dexamethasone. Anaesthesist.

[6] Tsurufuji S, Sugio K, Takemasa F (1979). The role of glucocorticoid receptor and gene expression in the anti-inflammatory action of dexamethasone. Nature.

[7] Barnes PJ (2006). How corticosteroids control inflammation: Quintiles Prize Lecture 2005. Br J Pharmacol.

[8] Rosenfeld DM, Ivancic MG, Hattrup SJ (2006). Perineural versus intravenous dexamethasone as adjuncts to local anaesthetic brachial plexus block for shoulder surgery. Anaesthesia.

[9] Hussain N, Van der Langenbergh T, Semer C (2018). Equivalent analgesic effectiveness between perineural and intravenous dexamethasone as adjuvants for peripheral nerve blockade: a systemic review and meta-analysis. Can J Anaesth.

[10] Waldron NH, Jones CA, Gan TJ, Allen TK, Habib AS (2012). Impact of perioperative dexamethasone on postoperative analgesia and side-effects: systematic review and meta-analysis. Br J Anaesth.

[11] Batistaki C, Kaminiotis E, Papadimos T, Kostopanagiotou G (2017). A narrative review of the evidence on the efficacy of dexamethasone on postoperative analgesic consumption. Clin J Pain.

[12] Steinthorsdottir KJ, Awada HN, Abildstrøm H, Kroman N, Kehlet H, Kvanner Aasvang E (2020). Dexamethasone dose and early postoperative recovery after mastectomy: a double-blind, randomized trial. Anesthesiology.

[13] De Oliveira GS, Almeida MD, Benzon HT (2011). Perioperative single dose systemic dexamethasone for postoperative pain. A Meta-analysis of Randomized Controlled Trials. Anesthesiology.

[14] Corcoran T, Kasza J, Short TG, O'Loughlin E, Chan MT, Leslie K, Forbes A, Paech M, Myles P, ENIGMA-II investigators (2017). Intraoperative dexamethasone does not increase the risk of postoperative wound infection: a propensity score-matched post hoc analysis of the ENIGMA-II trial (EnDEX). Br J Anaesth.

[15] Kakodkar PS (2013). Routine use of dexamethasone for postoperative nausea and vomiting: the case for. Anaesthesia.

[16] Thangaswamy CR, Rewari V, Trikha A, Dehran M, Chandralekha (2010). Dexamethasone before total laparoscopic hysterectomy: a randomized controlled dose–response study. J Anesth.

[17] Piccioni F, Segat M, Falini S, Umari M, Putina O, Cavaliere L, Ragazzi R, Massullo D, Taurchini M, del Naja C, Droghetti A (2018). Enhanced recovery pathways in thoracic surgery from Italian VATS Group: perioperative analgesia protocols. J Thorac Dis.

[18] Walder B, Schafer M, Henzi I, Tramèr MR (2001). Efficacy and safety of patient-controlled opioid analgesia for acute postoperative pain. A quantitative systematic review. Acta Anaesthesiol Scan.

[19] Gustafsson UO, Scott MJ, Hubner M, Nygren J, Demartines N, Francis N, Rockall TA, Young-Fadok TM, Hill AG, Soop M, de Boer HD, Urman RD, Chang GJ, Fichera A, Kessler H, Grass F, Whang EE, Fawcett WJ, Carli F, Lobo DN, Rollins KE, Balfour A, Baldini G, Riedel B, Ljungqvist O (2019). Guidelines for Perioperative Care in Elective Colorectal Surgery: Enhanced Recovery After Surgery (ERAS®) Society Recommendations: 2018. World J Surg.

[20] Mesbah A, Yeung J, Gao F (2016). Pain after thoracotomy. BJA Education.

[21] Umari M, Carpanese V, Moro V, Baldo G, Addesa S, Lena E, Lovadina S, Lucangelo U (2018). Postoperative analgesia after pulmonary resection with a focus on video-assisted thoracoscopic surgery. Eur J Cardiothorac Surg.

